# Simultaneous lancet-free monitoring of alcohol and glucose from low-volumes of perspired human sweat

**DOI:** 10.1038/s41598-018-24543-4

**Published:** 2018-04-25

**Authors:** Ashlesha Bhide, Sriram Muthukumar, Amreek Saini, Shalini Prasad

**Affiliations:** 1 Department of Bioengineering, University of Texas at Dallas, 800 West Campbell Road, Richardson, TX 75080 USA; 2 Enlisense LLC, 1813 Audubon Pond way, Allen, TX 75013 USA

## Abstract

A lancet-free, label-free biosensor for simultaneous detection of sweat glucose and alcohol was demonstrated using zinc oxide thin films integrated into a nanoporous flexible electrode system. Sensing was achieved from perspired human sweat at low volumes (1–3 μL), comparable to ambient conditions without external stimulation. Zinc oxide thin film electrodes were surface functionalized with alcohol oxidase enzyme and with glucose oxidase enzyme towards developing an affinity biosensor specific to the physiological relevant range of alcohol comprising of 0–2 drinks (0–50 mg/dl) and physiologically relevant range of glucose ranging from hypo- to hyper-glycaemia (50–130 mg/dl) in perspired human sweat. Sensing was achieved by measuring impedance changes associated with alcohol and glucose binding onto the sensor interface using electrochemical impedance spectroscopy with a dynamic range from 0.01–200 mg/dl and a limit of detection of 0.01 mg/dl for alcohol in human sweat. Sensor calibration in synthetic sweat containing interferents (25–200 mg/dl) and comparison using regression and Bland-Altman analysis of sweat sensor performance was done with BACtrack^®^. Combinatorial detection of glucose and ethanol in perspired human sweat and comparison of sweat sensor performance with Accu-Chek^®^ blood glucose monitoring system that we expect would be relevant for pre-diabetics and diabetics for monitoring their glucose levels and alcohol consumption.

## Introduction

Alcohol consumption is prevalent in the United States, with an estimated 109 million Americans who drink alcohol. The effect of alcohol on causation or prevention of diabetes has been studied. It appears that there may be a U-shaped relationship between alcohol and type 2 diabetes such that there is a higher risk of developing diabetes with both low and high intake levels and a lower risk with moderate intake^1^. Moderate alcohol consumption was defined as ~5–30 g/day. This corresponds to about 0.5–2.5 drinks per day. Alcohol consumption in people with type 2 diabetes can result in hypoglycemia because of decreased gluconeogenesis, decreased glycogenolysis, and possibly reactive hypoglycemia in response to carbohydrate intake. However, most data are retrospective or observational, and hence there is an immediate and significant need to determine the long-term effects of alcohol consumption on glycemic control, self-management behaviors, and the complications of diabetes^2,3^. In this paper, we demonstrate the first technological proof of feasibility in using human sweat based biosensors towards combinatorial monitoring of alcohol and glucose content in perspired human sweat towards the larger goal of designing diagnostics wearables that can quantify and dynamically report both alcohol and glucose content from users. The use of wearable biosensing in health care in the form of diagnostics wearables is still in a stage of infancy, but market research from TrustMarque points toward 81% of respondents wanting more use of connected devices in the area of chronic disease management^4^. Wearable biosensing technologies that detect biomarkers from human sweat have evolved from this need of non-invasive monitoring of social lifestyle choices coupled with a view of chronic disease management. Human sweat is one such biomarker rich fluid containing valuable medical information that can be the key driver for developing sweat based point-of-care health management devices^5^. Sweat is preferred candidate for bodily fluid- based analysis amongst others such as serum, saliva, and urine due to the ease of gland stimulation, sample gathering, and analysis. Biosensors that monitor human sweat biomarkers are required to have wide dynamic range of response, robust sensitivity and specificity in the physiologically relevant range. Non-invasive sweat monitoring is one for the most researched areas in recent times^6^. Alcohol bio-sensing has gained attention in clinical and forensic analysis of bodily fluids, and in food and beverage industries [See Table S1]. Alcohol abuse and addiction are leading causes of violence, driving under influence causing fatal vehicle crashes, and poses a severe threat to the lives of other drivers. Blood alcohol content (BAC) is an accurate indicator of ingested alcohol in the human circulatory system but requires tedious procedures (e.g. gas chromatography) and trained personnel to analyze samples^7^. Breathalyzers are currently being used to indirectly estimate BAC through measurement of breath alcohol content (BrAC). The standard breath alcohol to blood alcohol ratio is 2100:1 is based on Henry’s law which estimates breath alcohol present in exhaled volume of air from the blood alcohol present in the bloodstream^8^. Breath alcohol is susceptible to other alcohol components present in the environment and hence generates false positives. About 1% of alcohol metabolized by the liver leaves the body through sweat through diffusion from the skin and secretion from eccrine glands^9^. Previous studies have established a correlation between transdermal alcohol content (TAC) and blood alcohol content (BAC)^10^. Alcohol consumption modifies glucose homeostasis exerting contradictory effects on blood glucose levels contingent on nutrition states. Diabetes Mellitus requires frequent monitoring of glucose levels with glucose being present in the range 0.1–50 mg/dl in human sweat^11^. Alcohol consumption in a fasting state induces hypoglycemia effecting the process of gluconeogenesis; the response to alcohol intake in a fed state is the development of hyperglycemic condition inhibiting the effects of insulin^7^. Turner *et al*. studied the effects of alcohol consumption on glucose levels next morning in Type I diabetes individuals. Moderate consumption of alcohol (~2 standard drinks) accompanied by intake of food increased chances of developing delayed, acute hypoglycemia after breakfast the following morning^12^. The premise of this paper is to demonstrate a lifestyle monitor based on sweat alcohol detection designed on a flexible platform towards building a wearable, point-of-care diagnostic for: (1) Glucose management to monitor blood glucose levels after consumption of alcohol (2) addressing the rising need for controlling alcohol induced fatalities and damages.

## Results and Discussions

The organization of this section organized is as follows: (1) Structural characterization and functionalization of the developed sensor for alcohol sensing; (2) Evaluation of sensor performance in synthetic sweat buffers through AC and DC step based techniques; (3) Evaluation of sensor performance and calibration in human sweat through AC based technique; (4) Sensor calibration in synthetic sweat containing interferents and comparison of sweat sensor performance with BACtrack^®^ S80 Pro breathalyzer; (5) Combinatorial detection of glucose and ethanol in human sweat and comparison of the sweat sensor performance with Accu-Chek® Nano SmartView blood glucose monitoring system.

### Structural characterization and functionalization of the developed sensor for alcohol sensing

**Figure 1:**
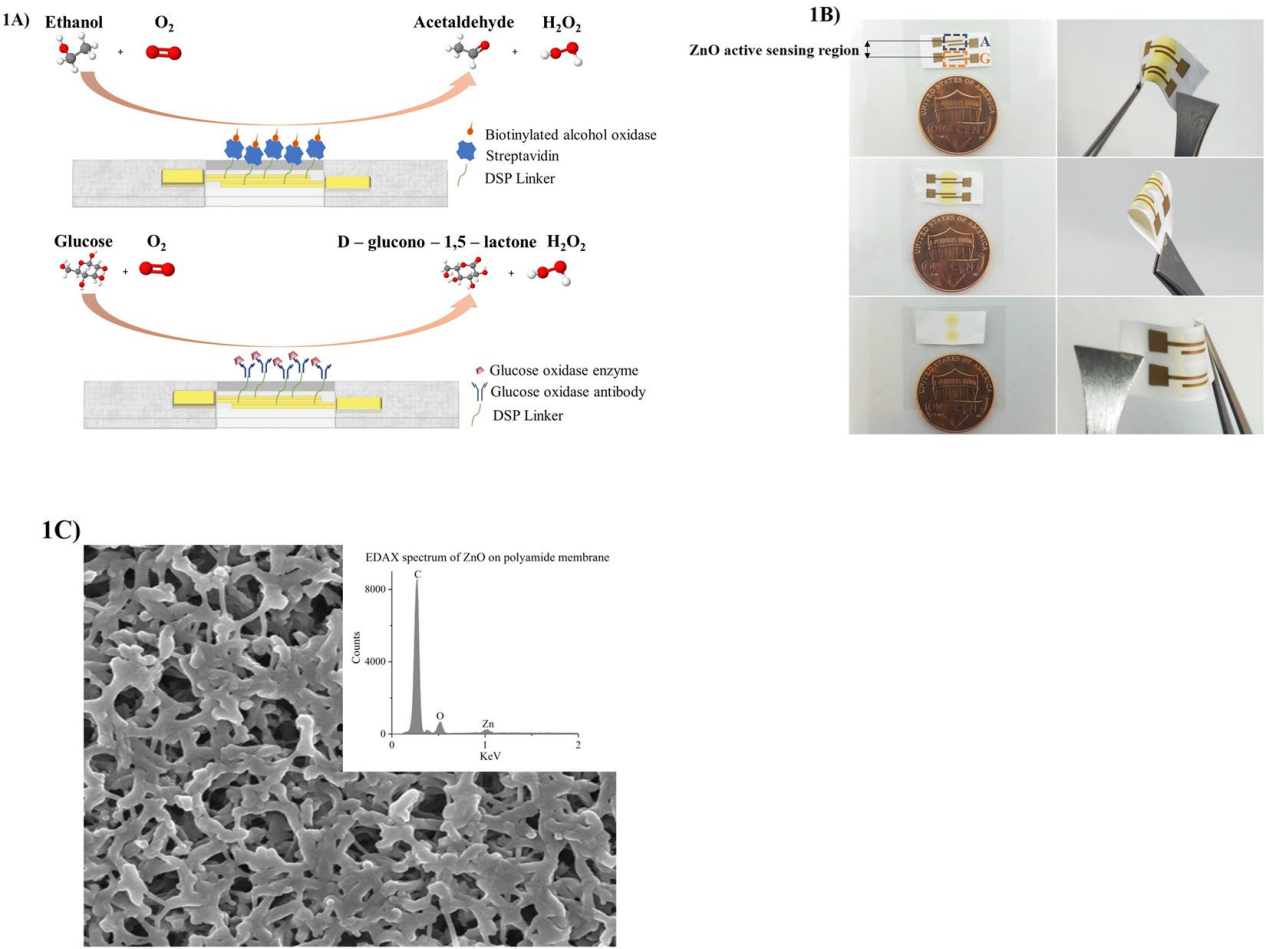
(**A**) Schematic of the immunoassay for the combinatorial detection of alcohol and glucose (**B**) Sweat sensor array showing fluid confinement in the active sensing region, sensor flexibility, and size comparison with one cent (**C**) SEM of polyamide membrane sputtered with ZnO. Inset shows the EDAX spectra of the sputtered membrane surface.

Human eccrine glands secret sweat at the rate 5–10 nl/min/gland^13^ typically which necessitates the use of low volumes for sensor functioning when it comes in contact with the skin. We use flexible, nanoporous polyamide membranes as the substrate material to fabricate the zinc oxide (ZnO) sensor stack. Analysis volumes as low as 1–3 µL are maintained through this study of biosensing in sweat buffers. Nanopores of the flexible substrate allow absorption of sweat molecules which are carried across to the sensor surface when sweat is introduced into the membrane. Affinity biosensing is primarily achieved through the capture probes bound to the active sensing region which interact with the biomolecules of interest as shown in Fig. 1A. Figure 1B shows the wicking of the 1–3 µL volume of the sweat on active sensing region on both faces of the sensor and sensor flexing when subjected to bending. The intercalated nanopore structures of the polyamide substrate act as a biomolecule sieve allowing only desirable biomolecules to travel to the sensor surface. Figure 1C shows the SEM image of ZnO sputtered on polyamide substrate with EDAX spectrum represented in the inset. The peak at 1KeV corresponds to the Zn L-shell of ZnO indicating the deposition of the film within the nanopores. Nanoconfinement of biomolecules based on size offers an enhanced signal to noise ratio (SNR) which is feasible for detecting small analytes because human sweat consists of ions and lipids that would otherwise contribute to the noise factor^14–17^. The binding events occurring within the nanopores in the vicinity of the electrode are reported in terms of impedance changes contributed by electrical double layer modulations and electron charge transfer.

### Evaluation of sensor performance in synthetic sweat buffers through AC and DC step based techniques

An AC based signal transduction technique electrochemical impedance spectroscopy is used to record the sensor response over a range of frequencies from 1 MHz to 1 Hz with a small AC excitation signal. This technique enables us to understand the charge modulation arising at the electrode-electrolyte interface as an outcome of biomolecular binding events and catalytic oxidation of alcohol by the enzyme occurring at the electrode surface. The interaction of a buffer with a semiconducting electrode surface modulates the capacitive reactance which is primarily driven through charge accumulation within the EDL. These dynamic changes occurring within the EDL at the electrode-electrolyte interface are derived from the imaginary impedance (Z_imag_) component characterized by EIS. Charge modulation in the EDL is affected by its Debye length (λ_D_) which is also a measure of the thickness of the double layer. The thickness of the Debye length is influenced by the ionic concentration of the buffer and hence dictates the charge screening effects at the electrode-electrolyte interface^18,19^.

**Figure 2:**
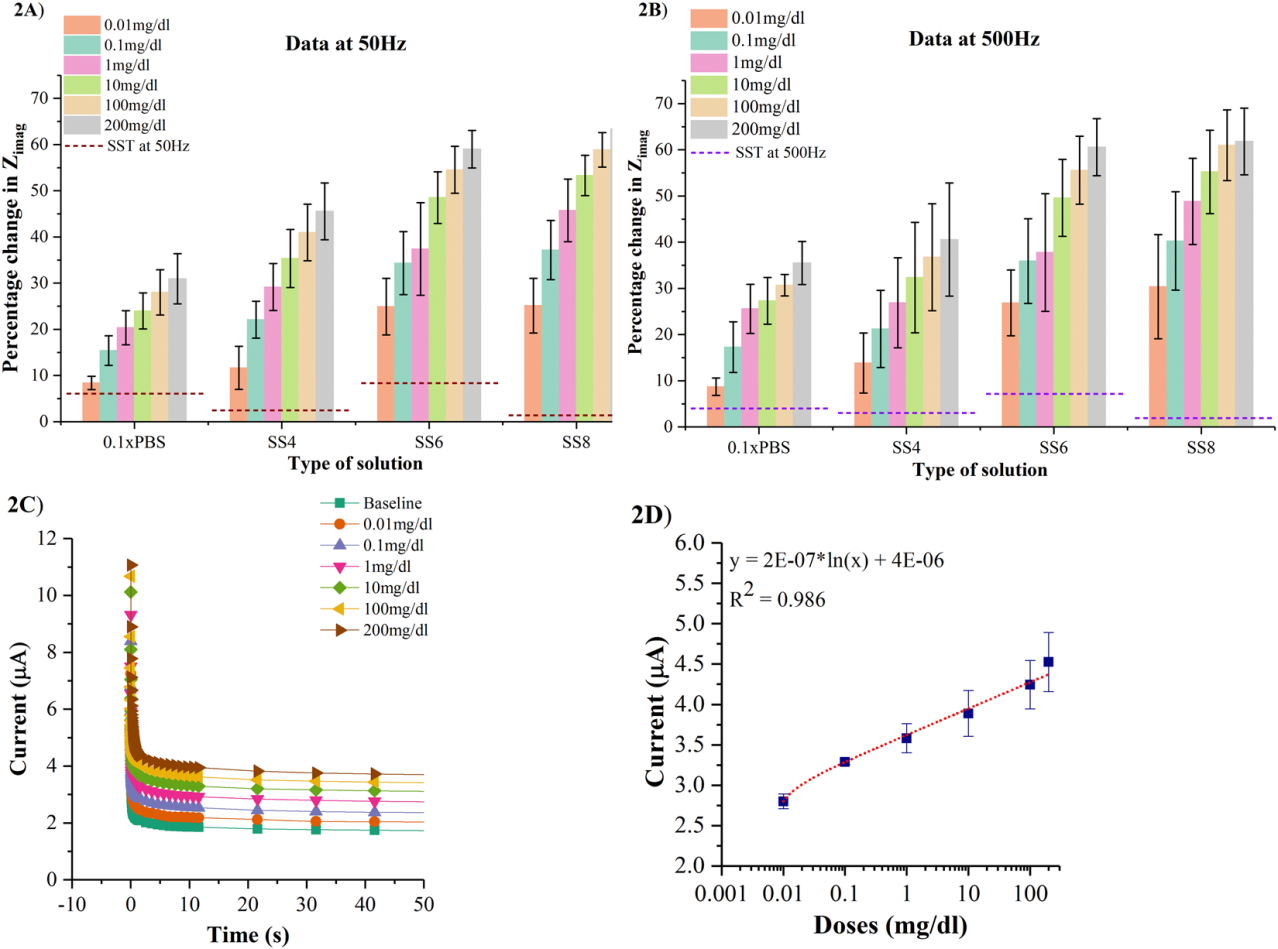
(**A**) Calibration dose response of ethanol in 0.1xPBS and synthetic sweat buffers of pH 4 to 8 as a function of percentage change in imaginary impedance (Z_imag_) at 50 Hz (**B**) Calibration dose response of ethanol in 0.1xPBS and synthetic sweat buffers of varying pH as a function of percentage change in imaginary impedance (Z_imag_) at 500 Hz (**C**) Chronoamperograms for ethanol concentrations in the range 0.01 mg/dl to 200 mg/dl spiked in SS of pH 6 (**D**) Calibration curve plotted for ethanol concentrations from 0.01 mg/dl to 200 mg/dl spiked in SS of pH 6 in terms of steady state current.

The responses of the sensor to varying ethanol concentrations in 0.1xPBS and synthetic sweat (SS) buffers of pH 4 to 8 captured in terms of percentage changes in imaginary impedance (Z_imag_) at 50 Hz and 500 Hz are shown in Fig. 2A,B respectively for n = 3 replicates. The percentage change in Z_imag_ is calculated as the variation in the impedance of the ethanol concentration response with respect to impedance of baseline measurement using the expression (100*(baseline Z_imag_ - spiked ethanol concentration Z_imag_)/baseline Z_imag_). A signal to noise (SNR) of 3 is chosen to compute the specific signal threshold (SST)^20^. SST is estimated by the expression (3*(σ_baseline_/µ_baseline_)). Control studies are performed by spiking 0.1xPBS with ethanol concentrations ranging from 0.01 mg/dl to 200 mg/dl that corresponds to the alcohol content present in perspired human sweat after consumption of 0 to 5 standard drinks. In this study, we chose to use 0.1 × PBS buffer (pH ~ 7.4) which has a λ_D_ of ~2.5 nm which is approximately equivalent to the size of biomolecules. Hence, the effect of charge screening on biosensing is minimized and a dose dependent impedance response was observed.

The percentage change in Z_imag_ for 0.01 mg/dl ethanol concentration in 0.1xPBS at 50 Hz and 500 Hz is ~8.5%. At 200 mg/dl ethanol concentration, the percent changes in Z_imag_ observed at 50 Hz and 500 Hz are ~30–35% respectively. The percentage impedance changes in Z_imag_ is distinguishable from its SST for the lowest ethanol concentration of 0.01 mg/dl which is identified as the limit of detection (LOD)^20^. The dynamic range of ethanol in 0.1xPBS is 0.01–200 mg/dl. Smaller percentage changes in Z_imag_ are observed in 0.1xPBS owing to the lower electrical conductivity of the buffer contributing to smaller charge accumulation changes^21^.

The percentage changes in Z_imag_ across the frequency range 50–500 Hz increase as the pH of synthetic sweat buffer varies from pH 4 to pH 8. At 0.01 mg/dl ethanol concentration, the percentage changes in Z_imag_ for SS of pH 4 in the range 50–500 Hz are ~11–13%. The percentage changes in Z_imag_ at 200 mg/dl ethanol concentration for SS of pH 4 in the range 50–500 Hz are ~40–45%. For SS of pH 6, the percentage changes in Z_imag_ for 0.01 mg/dl and 200 mg/dl ethanol concentrations is observed to be ~24–26% and ~60% respectively across the 50–500 Hz frequency range. In SS of pH 8, the percentage changes in Z_imag_ for 0.01 mg/dl ethanol concentration lie between ~25–30% for 50–500 Hz range while for 200 mg/dl ethanol concentration the percentage change is found to be ~63% in the frequency range 50–500 Hz. The LOD is found to be 0.01 mg/dl while the linear dynamic range (LDR) is preserved in the range 0.01–200 mg/dl for synthetic sweat buffers of pH range 4 to 8.

Heinonen *et al*. have demonstrated the stability of nanotextured ZnO films in acidic and basic conditions over an 8-week period^22^. We observed a higher response to ethanol dose concentration in synthetic sweat buffers of pH 6 and pH 8 than pH 4. The pH values of buffer solution favorable for a stable enzyme activity lie in the range 6.0–8.0^23,24^. Lower percentage changes in Z_imag_ at lower pH’s can also be attributed to the H^+^ ions from the surrounding buffer that interfere with the charge modulation in the EDL at the electrode-electrolyte interface^24^. The ZnO sensors used in this work can be operated over a frequency window of 50 Hz to 500 Hz as the percentage change in Z_imag_ is preserved for buffers over varying pH ranges allowing for the sensors to be interfaced with portable electronics and tuned as per their frequency operation range.

Chronoamperometry technique allows for the measurement of non-faradaic capacitive current generated at the electrode- electrolyte interface by applying a step dc potential. The chronoamperometric response curves, as shown in Fig. 2C for n = 2 replicates, represent the dose dependent changes in double layer capacitance through the charging currents in SS of pH 6. The current spike seen within ~1 s of the response is a result of non-faradaic charging of the electrical double layer following which there is an exponential decay in the current that is termed steady state current. The steady state current represents a linear change between 2.8–4.8 µA with increasing ethanol concentrations in the range 0.01–200 mg/dl and is represented as a calibration curve as shown in Fig. 2D. The sensitivity calculated from the calibration curve is 0.2 ± 0.02 µA/mM with the R^2^ of 0.986 which is comparable to the sensitivity reported by Kim *et al*. which demonstrates a wearable tattoo sensor based on the amperometric detection of alcohol in sweat using alcohol oxidase enzyme by using printed Prussian blue electrode^25^.

### Evaluation of sensor performance and calibration in human sweat through AC based technique

**Figure 3:**
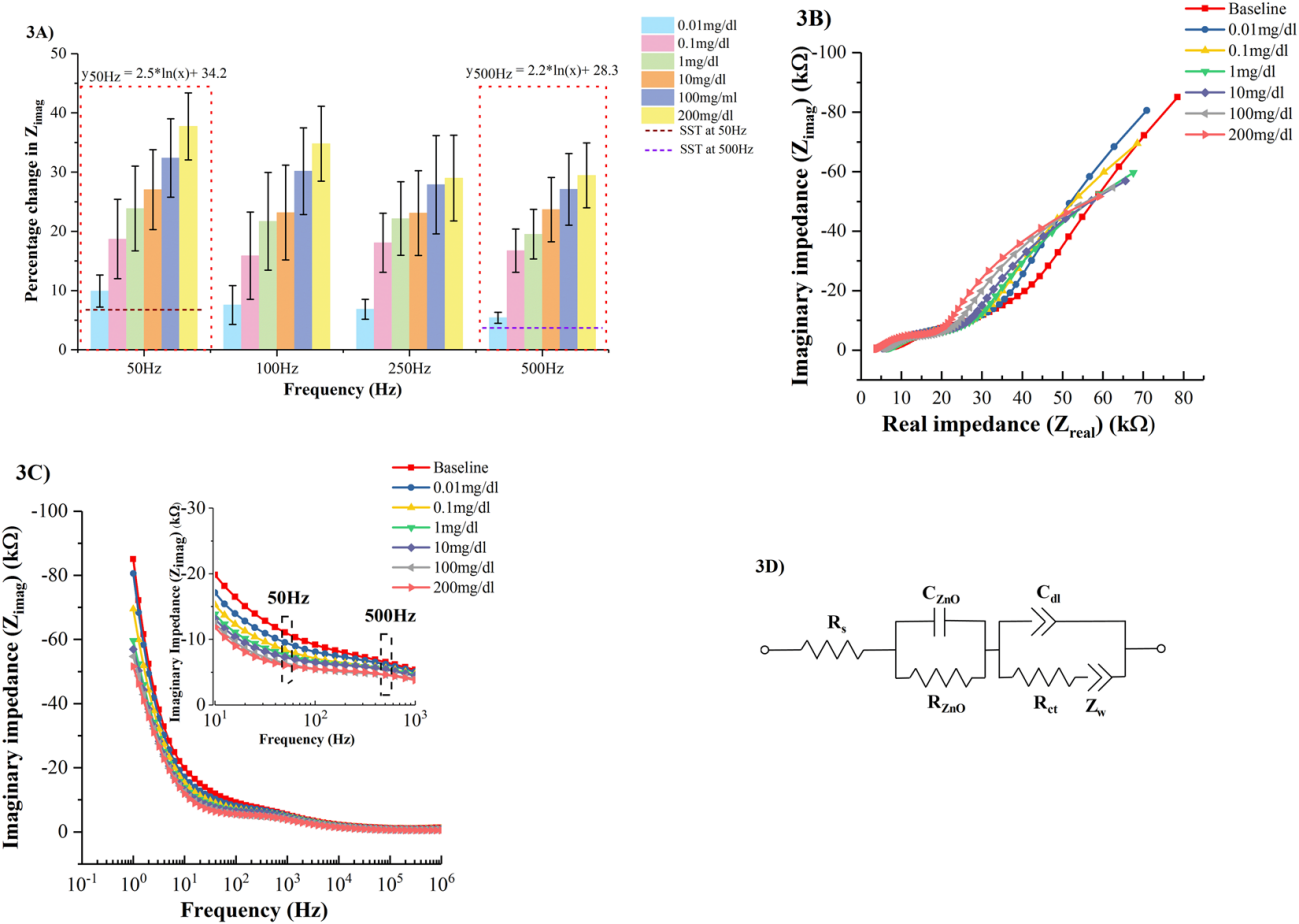
(**A**) Calibration dose response of ethanol in human sweat as a function of percentage change in imaginary impedance (Z_imag_) (**B**) Nyquist plots for ethanol concentrations 0.01–200 mg/dl spiked in human sweat (pH ~5.98) (**C**) Bode magnitude plots for ethanol concentrations 0.01–200 mg/dl spiked in human sweat (pH ~5.98). Inset shows the bode magnitude plots over the frequency range 10–1KHz (**D**) Equivalent circuit representation of the ethanol detection sweat sensor system.

The response of the sensor in human (pH ~5.98) sweat spiked with ethanol concentrations over the frequency window 50–500 Hz is shown in Fig. 3A for n = 6 replicates. The percentage change in Z_imag_ is analyzed over the frequency range from 1 Hz to 1 MHz. The outcome of this analysis is that highest signal-to-noise ratio is obtained over the frequency window 50–500 Hz with highest percentage change in Z_imag_ being observed at 50 Hz and the calibration trend being preserved across the frequency window. The sensor is sensitive within the chosen frequency window to capture the contributions from non- faradaic charge modulation. The same frequency window for evaluating the performance of the sensor in SS of pH 6. At 0.01 mg/dl, the percentage change in Zimag between 50–500 Hz is ~5–9%. The percentage change in Z_imag_ at 200 mg/dl ethanol within 50–500 Hz is ~30–34%. The SST for ethanol detection in human sweat is calculated to be ~5–7%. The LOD of ethanol in human sweat is measured to be 0.01 mg/dl. Higher standard deviation and lower percentage changes in Z_imag_ are observed in human sweat relative to SS of pH 6 because of contributions from interferents present in human sweat.

The Nyquist plot for ethanol detection in human sweat ranging from 0.01–200 mg/dl over frequencies 1Hz-1MHz is shown in Fig. 3B. The Nyquist plots can be divided into three regions. In the first region, real impedance (Z_real_) component varies from 3.7 KΩ to 8.7 KΩ and Z_imag_ varies from 500 Ω to 1.35 KΩ. This region corresponds to 1MHz–10KHz region on the Bode magnitude plots as shown in Fig. 3C. There is a shift in the semicircle towards left side on the x-axis of the plot with increasing ethanol concentration. This shift can be attributed to the change in the solution resistance as more ethanol molecules are catalytically oxidized in the presence of AOx forming reaction products. In the second region, corresponding to the mid frequency range 10–1 KHz in the Bode magnitude plot, Z_real_ varies 5.6–13.2 KΩ and Z_imag_ varies from 1.5–5 KΩ. This represents the capacitive and charge transfer properties of the semiconducting oxide film deposited on the nanoporous membrane. In the low frequency region between 1 KHz–10 Hz, Z_real_ varies between 14–34 KΩ while Z_imag_ varies between 4.1–15.4 KΩ. These changes reflect the binding events occurring within the EDL across the interface contributing to the variations in the capacitive reactance.

The equivalent circuit, as shown in Fig. 3D, is represented by a Randles circuit models a charged electrode surface in contact with an ionic buffer consisting of biomolecules of interest forms an EDL at the interface. The charge accumulation within the EDL is measured by the double layer capacitance (C_dl_); the solution resistance (R_s_) is a measure of the resistance of the bulk solution extending beyond the EDL; the electron transfer between the enzyme biomolecule complex and the electrode is represented by the charge transfer resistance (R_ct_). The first semicircle in the frequency range 1 MHz-10 KHz is a characteristic of the electrode material properties and is indicated as a parallel combination of resistor (R_ZnO_) and a capacitor (C_ZnO_). The second semicircle in the low frequency regime captures the changes within the EDL and is represented by a parallel combination of R_ct_ and C_dl_.

Using ZView^®^ circuit fitting analysis tool, the experimental impedance spectrum for ethanol dose concentrations in the range 0.01–200 mg/dl is fitted to the equivalent circuit shown in Fig. 3D and tabulated in Table 1. The double layer capacitance shows a dose dependent response validating a higher charge accumulation in the EDL with increasing dose concentrations as a result of more target ethanol molecules being catalytically oxidized.

**Table 1:** Extracted circuit fit parameters obtained by fitting the impedance response to the equivalent circuit.

Ethanolconcentrations (mg/dl)	Extracted circuit fit parameters				
	R_s_(KΩ)	R_ZnO_(MΩ)	C_ZnO_(µF)	R_ct_(KΩ)	C_dl_(nF)
Baseline	7.3	0.28	3.44	29.3	550
0.01	6.3	0.26	2.94	22.8	520
0.1	6.5	0.13	4.25	19.1	504
1	6.1	0.08	3.93	19.4	490
10	5.3	0.072	3.54	16.8	445
100	5.1	0.066	3.84	13.9	438
200	3.9	0.064	3.53	14.6	423

### Sensor calibration in synthetic sweat containing interferents and comparison of sweat sensor performance and BACtrack^®^ S80 Pro

**Figure 4:**
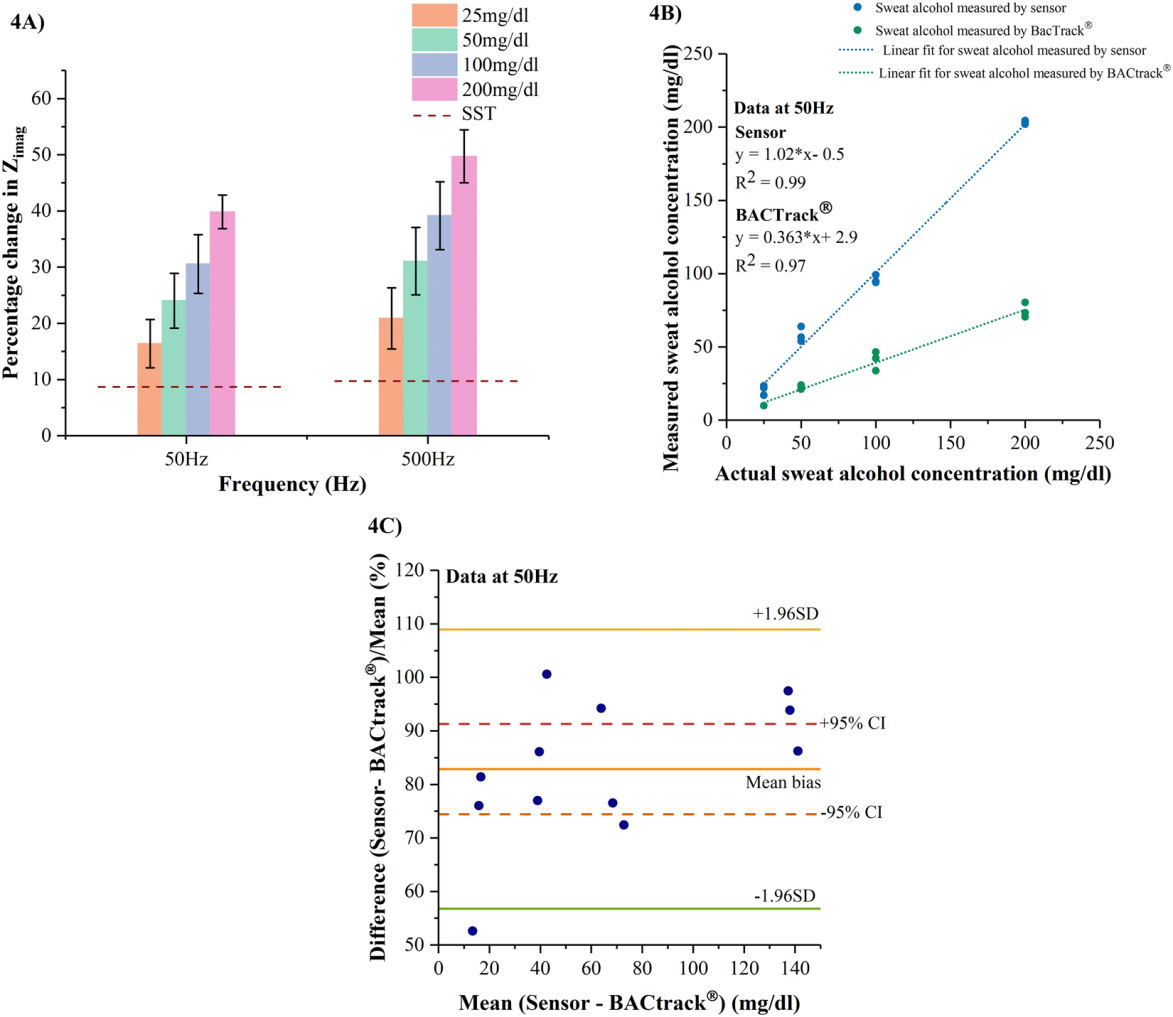
(**A**) Calibration dose response of ethanol spiked in SS of pH 6 with interferents as a function of percentage change in imaginary impedance (Z_imag_) (**B**) Regression plot for sweat ethanol measured by the sweat sensor at 50 Hz and BACtrack^®^ in comparison to the actual ethanol concentrations spiked in interferent based SS of pH 6 (**C**) Bland- Altman plot for the developed sweat sensor at 50 Hz compared to BACtrack®.

An interference study was carried out in SS of pH 6 (n = 3 replicates) to validate the selectivity of ethanol in the presence of interferents such as glucose, lactate, uric acid, ascorbic acid, and creatine. The calibration dose response for ethanol ranging from 25 mg/dl to 200 mg/dl present in the interferent spiked SS of pH 6 at 50 Hz and 500 Hz is shown in Fig. 4A. For 25 mg/dl ethanol concentration, the percentage change in Z_imag_ at 50 Hz and 500 Hz is ~25–32% whereas the percentage change in Z_imag_ for 200 mg/dl ethanol concentration across 50–500 Hz is ~45–55%. The LOD of ethanol in SS of pH 6 with interferents is 25 mg/dl. We observe an increasing percentage change in Z_imag_ with increasing ethanol dose concentrations as a results of increasing charge accumulation within the EDL. Here, the SST is established by the impedance response obtained in the absence of target ethanol molecules. The interferents study suggests the ability of the enzyme biomolecule complex to selectively react to ethanol molecules by neglecting the presence of interferents.

The performance of our sensor system in the presence of interferents is compared to a commercially available breathalyzer BACtrack® S80 Pro. Blood alcohol content (BAC) measured by BACtrack® is used to estimate sweat alcohol content (SAC) by the equation BAC (g/L) = 0.71*SAC (g/L)^10^. The responses of our sweat sensor and BACtrack® to sweat alcohol measured in the presence of interferents are correlated to the actual ethanol concentrations presented to both the systems through (i) Regression analysis to quantify the goodness of a fit between the ethanol concentrations measured by developed sweat sensor and BACtrack® in comparison to the actual ethanol spiked interferent-based sweat samples presented to them (ii) Bland-Altman analysis to quantify the agreement between the two measurement techniques. Regression analyses was carried out on n = 4 ethanol spiked synthetic sweat samples of pH 6 in the range 25–200 mg/dl with interferents as shown in Fig. 4B. In section 2, we have demonstrated alcohol sensing in synthetic buffers over the frequency window 50–500 Hz. We chose to present the outcomes of the statistical analyses at 50 Hz in this section. The results of the analyses at 500 Hz are displayed in supplementary in Figs S1and S2. R^2^ value of 0.99 is obtained for sweat sensor correlation at 50 Hz with the actual ethanol concentrations spiked in interferent based SS of pH 6. A significantly linear relationship can be drawn between the alcohol measured by the sweat sensor and the actual alcohol concentration dosed on it. We obtain an R^2^ of 0.97 for the correlation analysis done between BACtrack® and the actual dose concentrations presented to it. Although a linear fit is obtained between the actual and measured alcohol doses, a one to one agreement of the measurements is not seen between them. The linear fits obtained for both the sweat alcohol measurement techniques exhibit a linearly increasing offset with increasing ethanol dose concentrations. The offset observed in measuring the sweat alcohol content using BACtrack® is mainly because breathalyzers are designed to estimate BAC from a breath sample. We have simulated a breath like environment by nebulizing ethanol samples spiked in synthetic sweat of pH 6 with interferents^26^ [See Methods section]. The differences in analysis volumes utilized on both sensing platforms is another source of this offset. Figure 4C displays the Bland-Altman plots for sweat sensor compared to BACtrack® highlighting the bias and variations associated with the sensor and BACtrack®^27^. The normalized difference between sensor and BACtrack® is computed to address the variability in the differences of both measurement techniques. A mean bias value of 82.86% indicates that on an average the sensor estimates the ethanol concentrations accurately. All measurements except one lie within ±1.96 SD of the mean bias and are spread equally on either side of the bias. We find six measurements lying within the ±95% CI [74.4–91.3%] with minimum of one measurement per ethanol concentration to be in these limits.

### Combinatorial detection of ethanol and glucose in human sweat and comparison of the sensor performance and Accu-Chek^®^ Nano SmartView blood glucose monitoring system

**Figure 5:**
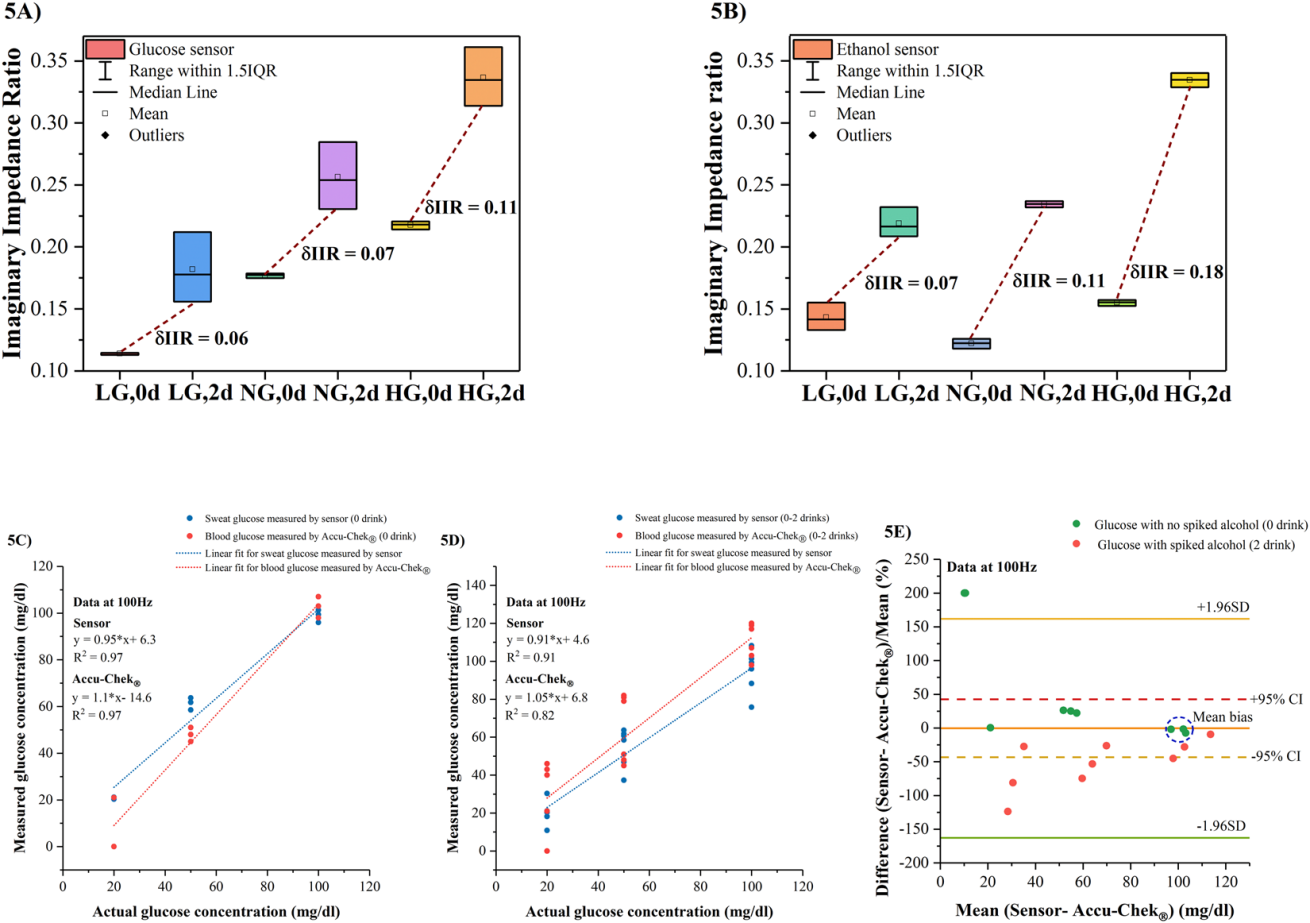
(**A**) Box plots for combinatorial detection of glucose levels in the presence of alcohol (0–2 standard drinks) as an interferent in human sweat on a glucose sensor at 100 Hz (**B**) Box plots for combinatorial detection of alcohol (0–2 standard drinks) in the presence of glucose as an interferent in human sweat on an alcohol sensor at 100 Hz (**C**) Regression plot for human sweat glucose measured by the sweat sensor at 100 Hz and blood glucose measured by Accu-Chek^®^ Nano SmartView glucose monitoring system in comparison to the actual glucose concentrations presented to them without alcohol (0 drink) (**D**) Regression plot for human sweat glucose measured by the sweat sensor at 100 Hz and blood glucose measured by Accu-Chek^®^ Nano SmartView glucose monitoring system in comparison to the actual glucose concentrations presented to them with and without alcohol (0–2 drinks) (**E**) Bland- Altman analysis for sweat sensor at 100 Hz and Accu-Chek^®^ for human sweat samples with varying glucose levels in the absence (0 drink) and presence of alcohol (2 drinks) at 100 Hz.

In this section, we present the results of our study on glucose and alcohol level modulations when combined in human sweat and detected on the developed glucose and alcohol sensing systems. Previously, correlations between blood glucose and sweat glucose levels have been established which are utilized in this study^28–30^. A dose combination index table, shown in Table 2, consists of glucose and ethanol concentrations used to make the concoctions for this study. The impedance response to the dose combinations is presented as an imaginary impedance ratio (IIR) calculated relative to the baseline Z_imag_. A specific analysis frequency of 100 Hz for the combinatorial detection is chosen from previous work^30^ which reported glucose detection in human sweat using the same sensor platform. The LOD and dynamic range for glucose detection in human sweat was reported to be 0.1 mg/dl and 0.01–200 mg/dl respectively. The sensitivity of glucose molecules to the assay was recorded as percentage changes in real and imaginary impedances which were attributed to the electron charge related to the biomolecule complex and ZnO sensor surface. The combinatorial effects of detecting a glucose levels in the presence of alcohol as an interferent in human sweat on a glucose sensor is depicted as a box plot in Fig. 5A for n = 3 samples. The average IIR varies from 0.11 to 0.21 with increasing glucose levels from 20–100 mg/dl in the absence of alcohol with a tighter spread in the IIR’s for increasing glucose levels. The average IIR for increasing glucose levels in the presence of alcohol (~2 standard drinks) as an interferent varies from 0.18–0.33 [See Table S2]. The changes in IIR (δIIR) between the glucose levels in the absence and presence of alcohol lie in the range 0.06–0.1. The shift and spread in IIR’s for glucose levels in the presence of alcohol is a manifestation of background noise from human sweat, cross talk between the target molecules, and variations in the molarities of the target concentrations while combining them into a concoction.

**Table 2:** Dose combination index.

Dose combination index			
Dose combinations	Labels	Glucose concentration (mg/dl)	Ethanol concentration (mg/dl)
Low glucose level, zero drink	LG,0d	20	0
Low glucose level, two drinks	LG,2d	20	50
Normal glucose level, zero drink	NG,0d	50	0
Normal glucose level, two drinks	NG,2d	50	50
High glucose level, zero drink	HG,0d	100	0
High glucose level, two drinks	HG,2d	100	50

For this study, we chose to represent the IIR at 100 Hz to draw a comparison between the combinatorial detection of glucose and alcohol on a glucose sensor in relation to an alcohol sensor. A box plot representation for the combinatorial detection of alcohol (0–2 drinks) in the presence of glucose at 100 Hz for n = 3 samples as shown in Fig. 3B. The average IIR range recorded by the sensor for a concoction consisting of only the interferent glucose in the absence of the target ethanol is 0.12–0.15; the IIR range recorded by the sensor for a concoction containing both the interferent and the target ethanol concentration (~2 standard drinks) is 0.21–0.13[See Table S2]. The modulation in the IIR in the presence of target molecules represented by the greater δIIR changes in the range 0.07–0.18 indicate the robustness of the alcohol assay in the presence of non-specific molecules. Higher impedance ratios observed in the presence of alcohol (~2 standard drinks) show a clear distinction in the frequency responses of the alcohol sensor in the absence and presence of alcohol.

Performances of the developed sweat glucose sensor and the commercially available Accu-Chek^®^ Nano SmartView blood glucose monitoring system are gauged with relative to the actual glucose concentrations presented to both systems. The correlation of the two measurement techniques with respect to actual glucose concentrations dosed is quantified by (i) R^2^ value and (ii) Bland-Altman analysis. Figure 5C represents the correlation analysis performed on sweat sensor and Accu-Chek^®^ using glucose samples (n = 3) without alcohol. Human sweat and blood samples are spiked with varying glucose and alcohol concentrations on the developed and the commercial sensor respectively as per the dose combinations shown in Table 2. An R^2^ of 0.97 for sweat and blood glucose samples without ethanol measured by both the systems relative to the actual sweat glucose concentrations dosed on sensor was determined. At 100 mg/dl glucose concentration both the systems overlap. At 50 mg/dl glucose concentration, repeat measurements lie in proximity to each other with small deviations observed for the sweat sensor and Accu-Chek^®^ respectively. For a low glucose concentration of 20 mg/dl, only one blood glucose measure of the triplicate was recorded by the blood glucometer while the sensor recorded sweat glucose levels accurately with a ± 1 mg/dl deviation. Similar correlation analysis is performed on sweat sensor and Accu-Chek^®^ to record their responses to glucose concentrations (n = 3) in the presence of alcohol and is represented in Fig. 5D. R^2^ values of 0.91 and 0.82 are observed for the sweat sensor and Accu-Chek^®^ correlation respectively. The skew in the correlation can be attributed to the ethanol molecules that hamper the glucose measurements. The glucose sensor system developed is efficient in neglecting the effects of interferent ethanol molecules. According to ISO15197 standards, it is acceptable for commercial glucometers to show a ±15 mg/dl deviation in measuring glucose concentration <75 mg/dl while for glucose concentrations ≥100 mg/dl, the acceptable deviation is only about ±5 mg/dl^31^. The differences between the developed sweat glucose sensor and Accu-Chek^®^ Nano SmartView glucose monitoring system can be better inspected from the Bland-Altman plots as shown in Fig. 5E. The mean bias for both the measurement techniques is −10 mg/dl or −0.39% indicating that differences between them are subtle and not significant. For sweat and blood glucose measurement performed in the absence of alcohol, 70% of the measurements lie within ±95% CI. At 100 mg/dl glucose concentrations, the mean differences (circled by blue in Fig. 5E) between developed sensor and Accu-Chek^®^ meet the ISO standards of measurement accuracy at glucose concentrations beyond 75 mg/dl. Mean differences at 50 mg/dl glucose concentration vary by 25% from the mean bias owing to drift at low concentrations. Two measurements at 20 mg/dl glucose concentration lying out of the limits of agreement due to the blood glucometer not being able to detect very low concentration and are recorded as zero. The alterations in the performances of the sensor and Accu-Chek^®^ in the presence of ethanol (~2 drinks) is evident in the Bland-Altman plot. Triplicate measurements done at 20 mg/dl glucose concentration exhibit a high degree of variability contributed by the interferent, sensor drift, and inability to obtain readings from the glucometer.

## Conclusion

Wearable biosensing has provided a non-invasive platform to monitor metabolites in bodily fluids such as human sweat to track fluctuations in glucose levels on consumption of alcohol as a part of social drinking in diabetic individuals that makes it imminent to address the need of developing a patch sensor that can accurately and non-invasively measure alcohol and glucose. Transdermal alcohol content is correlated in magnitude to blood alcohol content but lags in response time with respect to blood alcohol content which is governed by the parameters that control ethanol transport through the skin^32^. Desirable characteristics of wearable biosensors that make them commercially viable are enhanced sensitivity, capability to detect low target biomolecules in low analysis volumes within clinically relevant ranges, low power consumption, and cost effectiveness. The novelty proposed in this paper is an electrochemical biosensor fabricated on a flexible, nanoporous substrate to combinatorial detect glucose levels reliably in the absence and presence of alcohol in human sweat. Such a device is of help for pre-diabetic and diabetic individuals to monitor their glucose levels and limiting their intake of alcohol for healthier lifestyle choices. The findings of this study are that we could distinguish the effects of alcohol (~2 standard drinks) on low, normal, and high glucose levels prior to and post consumption of alcohol in low analysis volumes of 1–3 µL. Standard agreement analyses as per Clinical and Laboratory standards institute (CLSI)^33^ such as regression and Bland-Altman analyses are used to compare the performance of the developed sensor in comparison to a commercially available Accu-Chek® blood glucometer. Electrical impedance spectroscopy is used to capture the impedance changes occurring at the interface of the sensor surface and the buffer containing the target biomolecules of interest. Biomolecule detection is effected by the ionic strength, pH and conductivity of the buffer. We demonstrated the performance of the sensor on detection of alcohol in changing pH levels of synthetic sweat and in the presence of other metabolites as interferents to show the selectivity of ethanol molecules to the immunoassay. The responses of the sweat sensor and a commercially available breathalyzer BACtrack^®^ to synthetic sweat solution containing interferents is correlated to the actual alcohol concentrations made available to both the systems through regression and Bland-Altman analysis. Alcohol detection in human sweat is demonstrated over a linear dynamic range of 0.01–200 mg/dl which is equivalent to 0–5 standard drinks with a lower detection limit of 0.01 mg/dl. The detection of ethanol molecules in terms of changes in the capacitive reactance component of the impedance measured can be attributed to the higher charge accumulation occurring within the EDL across the ZnO- sweat buffer interface and electron charge transfer occurring between the enzyme biomolecule complex and the ZnO surface. Nanoconfinement of target biomolecules within the pores of substrate reduces noise from other molecules present in the sweat matrix leading to a higher SNR and distinguishable impedance signal from background noise^14,34^.

## Methods

### Reagents and materials

Polyamide substrates with a pore size of 0.2 µm were obtained from GE Healthcare Life Sciences (Piscataway, NJ, USA). The linker molecule dithiobis-succinimidyl propionate (DSP) and its solvent dimethyl sulfoxide (DMSO) were purchased from Thermo Fisher Scientific Inc. (Waltham, MA, USA). Salt-free streptavidin from Streptomyces avidiini, alcohol oxidase enzyme from Pichia pastoris, glucose oxidase from Asperigillus niger, D-(+)- glucose, absolute ethyl alcohol (≥99.5%), and sodium bicarbonate (≥99.7%) were procured from Sigma- Aldrich (St. Louis, MO, USA). Sodium L-Lactate, Creatinine, L- Ascorbic acid, and Uric acid were obtained from Sigma-Aldrich (St. Louis, MO, USA). Biotin (Long arm) NHS was purchased from Vector laboratories (Burlingame, CA, USA). The antibody for glucose oxidase was obtained from Abcam (Cambridge, MA, USA). The antibody for glucose oxidase enzyme was diluted in 1× phosphate buffered saline (PBS, Thermo Fisher Inc., Waltham, MA, USA). Streptavidin was lyophilized in 1× PBS and biotin was dissolved in DMSO. Synthetic sweat was prepared as per the recipe described in Table 2 of M.T. Mathew *et al*.^35^. The pH range was varied by varying the concentrations of the constituents. Single donor human sweat of pH ~6 was purchased from Lee Biosolutions Inc. (Maryland Heights, MO, USA). No preservatives were added to this product and it was stored at below −20 °C. Anticoagulated venous or arterial blood sample drawn from a patient was obtained from Carter BloodCare (Plano, TX, USA). Experiments were performed within two days of obtaining the blood samples. All ethanol dilutions are made in synthetic and human sweat. All glucose dilutions were made in human sweat. 0.1 × PBS was made by diluting PBS in deionized water (conductivity 18.5 MΩ.cm). BACtrack^®^ S80 Pro was purchased from Amazon.com, Inc. (Seattle, WA, USA). MQ5600 Nebulizer machine system was purchased from Mountainside Medical Equipment (Marcy, NY, USA). Accu-Chek^®^ Nano SmartView blood glucose monitoring system and the test strips were purchased on the counter from CVS pharmacy (Plano, TX, USA).

### Sensor fabrication

Figure 1B shows the sensor stack deposited on nanoporous polyamide substrate. The sensor stack consists of gold electrodes and a ZnO active sensing region. Gold electrodes are deposited using a shadow mask in a Temescal e-beam evaporator tool (Ferro Tec, Livermore, CA, USA). ZnO thin films are sputtered using AJA Orion RF magnetron with a 99.999% ZnO target (Kurt J. Lesker) at room temperature. The thickness of the film is measured to be ~100 nm using a Veeco Dektak 8 profilometer.

### Structural characterization

The deposition of the ZnO thin film on the flexible nanoporous substrate is investigated and characterized through SEM images captured using SUPRA SEM (Zeiss, Oberkochen, Germany).

### Sensor calibration for alcohol detection in synthetic and human sweat

The immunoassay protocol followed for the detection of alcohol in synthetic and human sweat is illustrated in Fig. 1A. 10 mmol of DSP linker is diluted in DMSO and functionalized on the sensor surface by dispensing 1–3 µL volume for 3 hours. A PBS wash is carried out prior to incubation of streptavidin for 1 hour on the sensor surface [See Fig. S3]. Another PBS wash is given post immobilization of streptavidin following which biotinylated alcohol oxidase enzyme is immobilized on the sensor surface by incubating for 15 minutes [See Fig. S4]. Alcohol oxidase enzyme is biotinylated as per the recipe formulated by Du *et al*.^36^. Synthetic sweat of desired pH is dosed on the sensor prior to introducing the doses and is considered as the baseline or the zero-dose step. Dilutions of ethanol made in synthetic sweat in the increasing range from 0.01–200 mg/dl are dispensed on the sensor and incubated for 10 minutes. EIS measurements record the current flow taken using a potentiostat (Gamry Instruments, Warminster, PA, USA) after an AC excitation signal with a frequency sweep of 1 Hz to 1 MHz is applied. All measurements are carried out in dark and under ambient temperature conditions. Similar protocols are followed for detection of alcohol in human sweat and 0.1 × PBS buffer solutions. Chronoamperometry (CA) measurements are performed using the same potentiostat after applying a constant step voltage of 200 mV for 60 seconds. CA measurements are done for the same ethanol doses in synthetic sweat buffer of pH 6 to match the pH of human sweat (~6).

### Sensor performance for alcohol detection in synthetic sweat with interferents and comparison with BACtrack^®^ S80 Pro

Synthetic sweat of pH 6 is spiked with metabolites present in sweat by making additions of 5.6 µM glucose, 3.7 mM lactate, 8.8 µM creatinine, 10 µM ascorbic acid, and 4.2 µM uric acid^37^. All ethanol dilutions are made in interferent spiked SS of pH 6 in the range from 25 mg/dl to 200 mg/dl and dispensed on the sensor. 6 mL analysis volume of the same ethanol concentrations in the interferent spiked SS of pH 6 is used to nebulize the solution to mimic breath. The nebulized solution is passed into the mouthpiece of BACtrack® through a simple adapter system that connects the nebulizer output line to the mouthpiece of BACtrack® which further outputs BAC (%) readings corresponding to the concentration of ethanol solution used. The BAC (%) readings are converted into SAC (mg/dl) as per the equation mentioned in section 4.

### Combinatorial detection of glucose and ethanol in human sweat and comparison of sensor performance with Accu-Chek^®^ Nano SmartView blood glucose monitoring system for samples in the absence and presence of alcohol

Combinatorial detection of glucose and alcohol is done by preparing a concoction of glucose and ethanol in human sweat. Glucose is spiked in concentrations of 20 mg/dl, 50 mg/dl, and 100 mg/dl in human sweat. Ethanol is spiked simultaneously in concentration of 50 mg/dl in human sweat. Immunoassay protocol for glucose detection is followed as per the protocol outlined in Munje *et al*.^30^. The glucose oxidase and alcohol oxidase enzyme biomolecule complexes are used on two different sensors deposited on the same substrate as shown in Fig. 1B. Combined concentrations of glucose and ethanol in the order as mentioned in Table 2 are dispensed in 1–3 µL volume on the glucose and alcohol sensors individually in the regions depicted by G and A symbols in Fig. 1B. EIS measurements post dose incubations are obtained in a similar manner with similar parameters as mentioned above. Test samples for blood glucose measurement using the Accu-Chek® blood glucose meter were prepared by spiking blood with glucose and ethanol concentrations are mentioned in Table 2. n = 3 readings are obtained for every dose combination. No animal or human subjects testing was performed during data collection for this manuscript.

### Statistical analyses

All the data is analyzed using OriginPro. Data is presented as mean ± standard error of mean (SEM) throughout this manuscript. SEM is calculated for the number of replicates or repeats used for experimentation and are mentioned in the results section. Error bars are plotted in terms of SEM.

### Data availability

Data generated and analyzed during this study is available from the corresponding author on request.

## Electronic supplementary material

Supplementary Information
