# Supplementary material for: Simultaneous lancet-free monitoring of alcohol and glucose from low-volumes of perspired human sweat
Source: Sci Rep. 2018 Apr 25;8:6507. doi: 10.1038/s41598-018-24543-4 (PMC5916883; doi:10.1038/s41598-018-24543-4)
Supplement: Supplementary file 1 — Supplementary Information [file 41598_2018_24543_MOESM1_ESM.doc]

**Simultaneous lancet-free monitoring of alcohol and glucose from low-volumes of perspired human sweat**

Ashlesha Bhide^1^, Sriram Muthukumar^2^, Amreek Saini^1^ and Shalini Prasad^1, †^

^1^Department of Bioengineering, University of Texas at Dallas, Richardson, TX 75080

^2^EnLiSense LLC, 1813 Audubon Pond Way, Allen, TX 75013

^†^Corresponding author, Email: [shalini.prasad@utdallas.edu](mailto:shalini.prasad@utdallas.edu)

**Supplementary information**

Supplementary table S1. Comparison of currently available enzymatic alcohol biosensors over past 10 years

| **Substrate** | **Analyte** | **Recognition element** | **Transduction**  **mechanism** | **Advantages** | **Limitations** | **References** |
| --- | --- | --- | --- | --- | --- | --- |
| **Screen printed carbon electrodes** | Ethanol in human sweat | Pichia pastoris alcohol oxidase enzyme | Amperometry | Non-invasive, wearable, real- time wireless on- body alcohol monitoring, | Iontophoresis requires skin agitation | 3 |
| **Polyaniline** | Ethanol for halal verification in fermented beverages | Pichia pastoris alcohol oxidase enzyme | Visual color change | pH 6.0- 8.0 distinguished,  LOD of 0.001% (v/v) ethanol,  Response time of 5s | Qualitative detection | 4 |
| **PPy, PEDOT, PEDOP** | Ethanol in distilled beverages | Pichia pastoris alcohol oxidase enzyme | Amperometry | PEDOT- highest affinity,  PEDOP- highly stable | Effected by interferents, Maximum sensor activity within small pH range of 6.5- 7.5 | 5 |
| **PVA- MWCNT-ADH bio- composite** | Ethanol in alcoholic beverages | Alcohol dehydrogenase alcohol oxidase enzyme | Cyclic voltammetry, Amperometry | Sensitivity – 196nA mM^-1^, Linear range – 1.5mM, Response time of 8s | Requires addition of coenzyme NAD^+^,  0.6V applied,  Long time operation not possible reduced lifetime of the enzyme | 6 |
| **Gold modified by non-conductive PPy** | Alcoholic grade determination in beverages | Pichia pastoris alcohol oxidase enzyme | Amperometry | First generation interference- free biosensor,  Sensitivity- 4.1mA M^-1^cm^-2^,  Operational stability, LOD – 2.3uM | Non-flexible, +1.5V applied, complex sensor fabrication process | 5 |

The intent of this table is to compare alcohol biosensors developed in recent times. Most of the biosensors were developed to determine the alcoholic content in beverages. Kim et al. demonstrated the first sweat based biosensor for alcohol determination in human sweat post alcohol consumption.


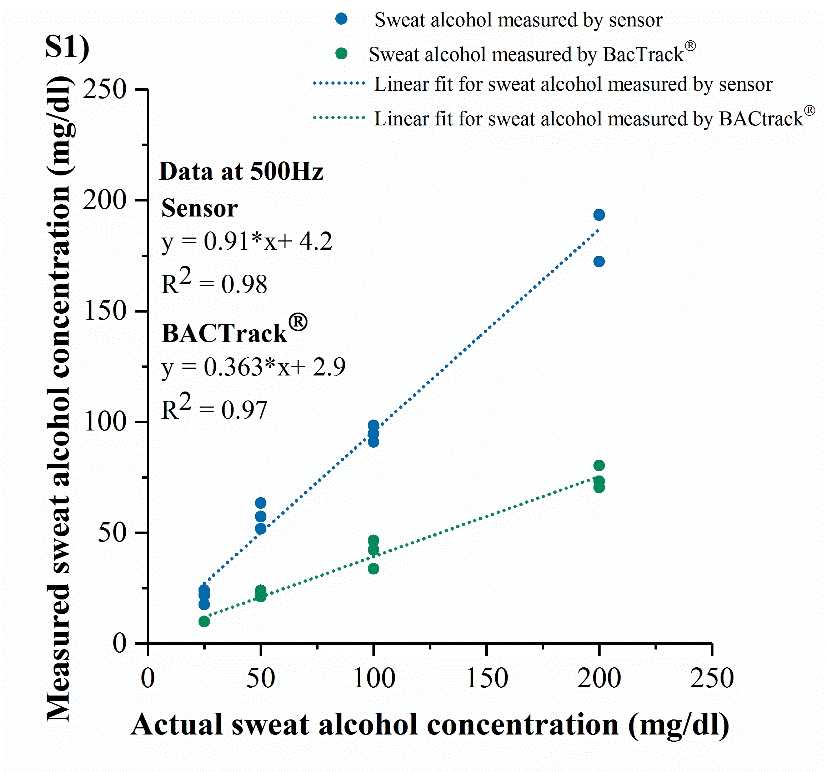
Figure S1. Regression plot for sweat ethanol measured by the sweat sensor at 500Hz and BACtrack® in comparison to the actual ethanol concentrations spiked in interferent based SS of pH 6

R^2^ value of 0.98 is obtained for sweat sensor correlation at 500Hz with the actual ethanol concentrations spiked in interferent based SS of pH 6. An R^2^ of 0.97 is obtained for the correlation analysis done between BACtrack® and the actual dose concentrations. A linear relationship is obtained between the alcohol measured by the sweat sensor and the actual alcohol concentration dosed on it. The linearly increasing deviations in the correlation curves produced by the sweat sensor and BACtrack® are from the measurement offsets obtained from BACtrack®.


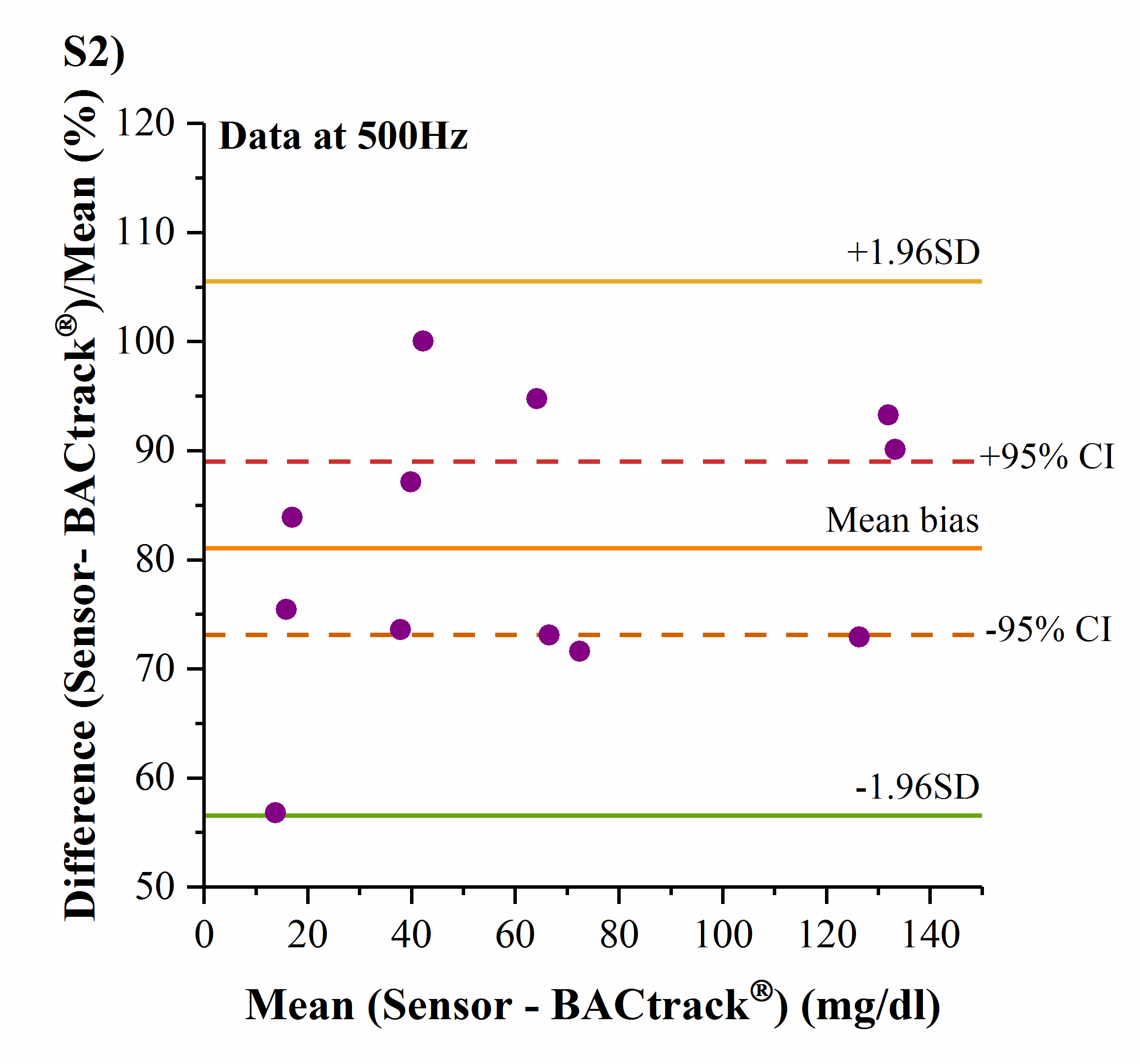
Figure S2. Bland- Altman plot for the developed sweat sensor at 500Hz compared to BACtrack®.

A mean bias value of 81.05% indicates that on an average the sensor estimates the ethanol concentrations accurately. All measurements except one lie within ±1.96SD of the mean bias and are spread equally on either side of the bias. Six measurements lie within the ±95% CI [73.11– 83.9%] with minimum of one measurement per ethanol concentration to be in these limits. The statistical analyses performed at both 50Hz and 500Hz produce comparable results allowing for sensor usability within a wide frequency window.

Supplementary table S2. Raw imaginary impedance ratios obtained for combinatorial detection of glucose and alcohol in human sweat.

| **Dose Combinations** | **Imaginary impedance ratio (IIR) for glucose sensor** | **Imaginary impedance ratio (IIR) for ethanol sensor** |
| --- | --- | --- |
| LG0d | 0.12 | 0.16 |
| LG0d | 0.12 | 0.15 |
| LG0d | 0.12 | 0.14 |
| LG2d | 0.22 | 0.24 |
| LG2d | 0.18 | 0.22 |
| LG2d | 0.16 | 0.21 |
| NG0d | 0.18 | 0.12 |
| NG0d | 0.18 | 0.13 |
| NG0d | 0.18 | 0.13 |
| NG2d | 0.29 | 0.24 |
| NG2d | 0.26 | 0.24 |
| NG2d | 0.24 | 0.24 |
| HG0d | 0.23 | 0.16 |
| HG0d | 0.22 | 0.16 |
| HG0d | 0.22 | 0.16 |
| HG2d | 0.37 | 0.35 |
| HG2d | 0.34 | 0.34 |
| HG2d | 0.32 | 0.33 |


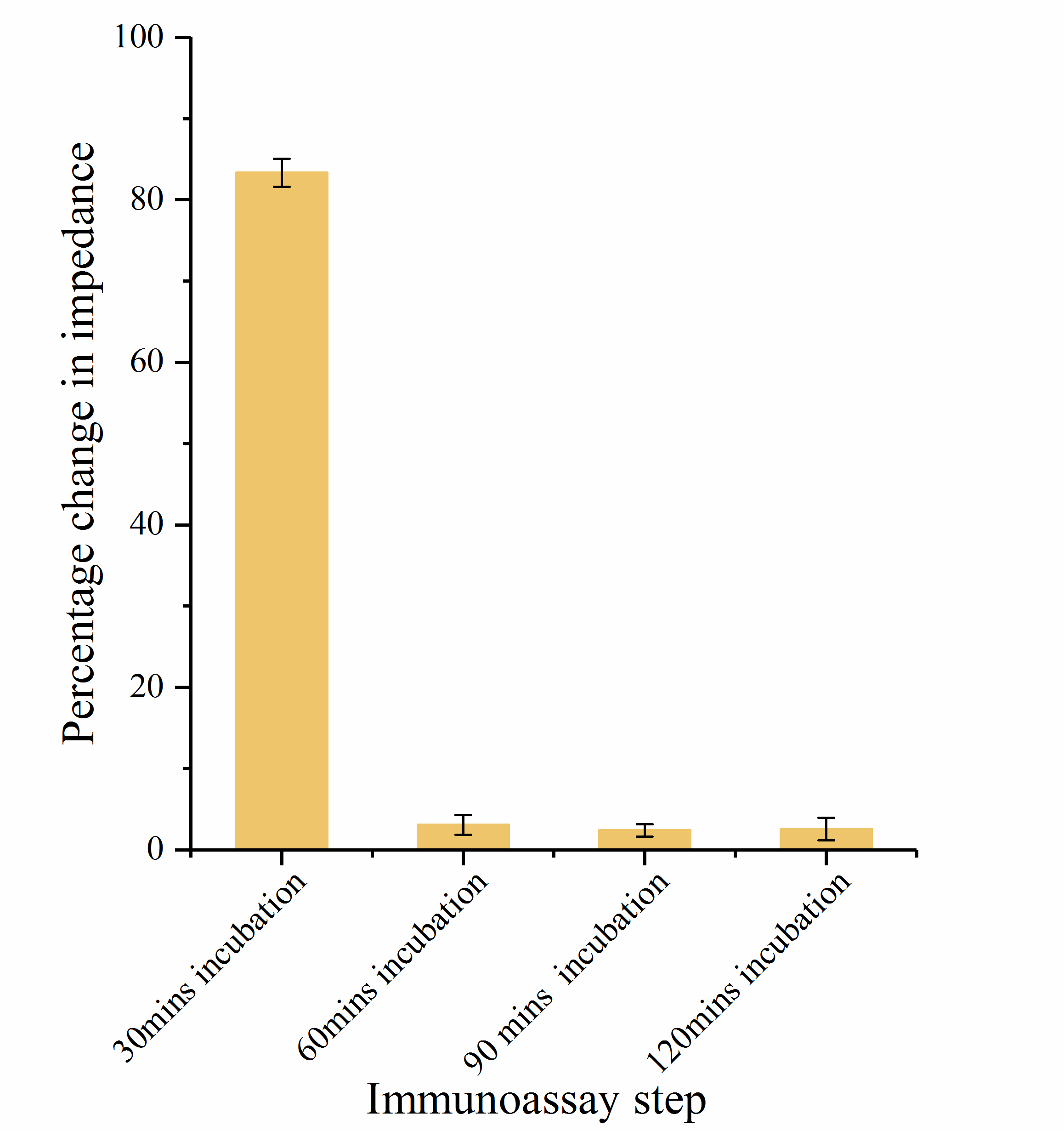


Figure S3. Streptavidin incubation time study

Streptavidin incubation time study (n= 3 replicates) was necessary to confirm the time required for streptavidin to bind to DSP linker. Percentage change in impedance is calculated relative to the impedance of the previous immunoassay step. 80% change in impedance after 30-minute incubation of streptavidin indicates the binding of streptavidin molecules to DSP molecules in progress. As shown in Fig.S3, the percentage changes in impedance between 60 to 120-minute incubation do not change significantly indicating that majority of streptavidin molecules are bound to DSP. Hence, a 60-minute incubation time is chosen.


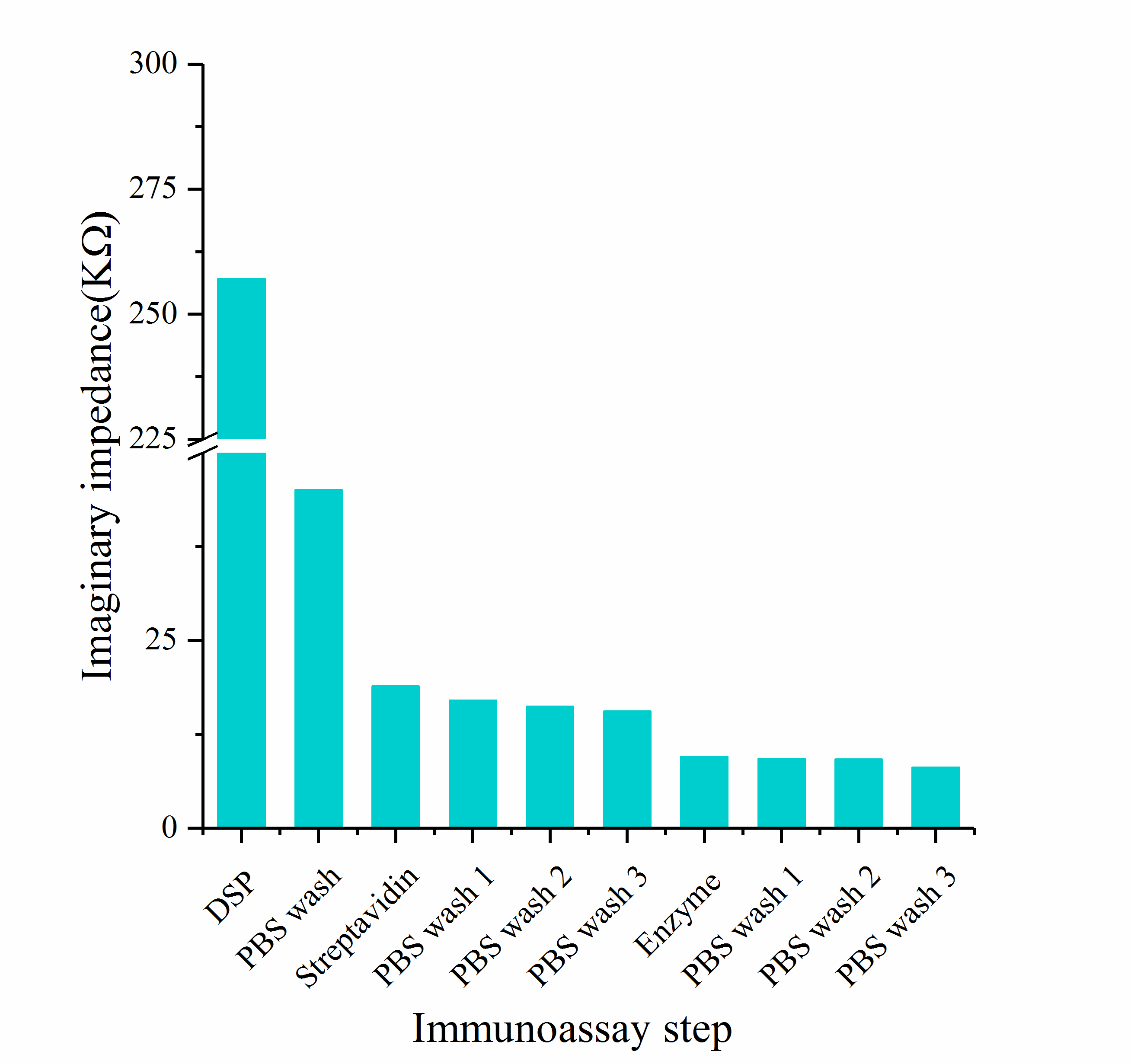
Figure S4. DSP, streptavidin, and biotinylated enzyme functionalization study

A confirmation study was carried to ensure the binding of biomolecules at various steps while building the immunoassay. The imaginary impedances at each step was recorded as shown in Fig. S4. Large impedance of 257 KΩ at DSP step is an indication of resistive nature of DSP in DMSO. PBS wash post DSP incubation is done to remove any unbound DSP molecules. A significant impedance change of 240KΩ from the DSP step is observed after one hour treatment with streptavidin indicating binding of streptavidin to the sensor surface saturated with DSP linker molecules. 3x PBS washes were carried out to remove any unbound streptavidin molecules. The impedance decreases to 9.4KΩ after functionalization of the biotinylated enzyme for 15 minutes. 3x PBS washes were carried after enzyme immobilization to remove any unbound enzyme molecules. No significant impedance changes are observed post 3x PBS washes verifying the biomolecular binding occurring at every immunoassay step. Decrease in impedance with each binding step is attributed to the formation of electrical double layer due to the increasing interaction of the biomolecules with the electrode.

**REFERENCES**

1. Kim, J. *et al*. Noninvasive Alcohol Monitoring Using a Wearable Tattoo-Based Iontophoretic-Biosensing System. *ACS Sens.* **1**, 1011-1019 (2016).

2. Kuswandi, B., Irmawati, T., Hidayat, A. M., Jayus & Ahmad, M. A Simple Visual Ethanol Biosensor Based on Alcohol Oxidase Immobilized onto Polyaniline Film for Halal Verification of Fermented Beverage Samples. *Sensors* **14** (2014).

3. Türkarslan, Ö, Böyükbayram, A. E. & Toppare, L. Amperometric alcohol biosensors based on conducting polymers: Polypyrrole, poly(3,4-ethylenedioxythiophene) and poly(3,4-ethylenedioxypyrrole). *Synthetic Metals* **160**, 808-813 (2010).

4. Tsai, Y., Huang, J. & Chiu, C. Amperometric ethanol biosensor based on poly (vinyl alcohol)–multiwalled carbon nanotube–alcohol dehydrogenase biocomposite. *Biosensors and Bioelectronics* **22**, 3051-3056 (2007).

5. Carelli, D., Centonze, D., De Giglio, A., Quinto, M. & Zambonin, P. G. An interference-free first-generation alcohol biosensor based on a gold electrode modified by an overoxidised non-conducting polypyrrole film. *Analytica Chimica Acta* **565**, 27-35 (2006).
